# Supplementary material for: Impedance Spectroscopic Study of Nickel Sulfide Nanostructures Deposited by Aerosol Assisted Chemical Vapor Deposition Technique
Source: Nanomaterials (Basel). 2021 Apr 24;11(5):1105. doi: 10.3390/nano11051105 (PMC8146325; doi:10.3390/nano11051105)
Supplement: Supplementary file 1 [file nanomaterials-11-01105-s001.zip › nanomaterials-1142548-supplementary.pdf]

Supplementary Information

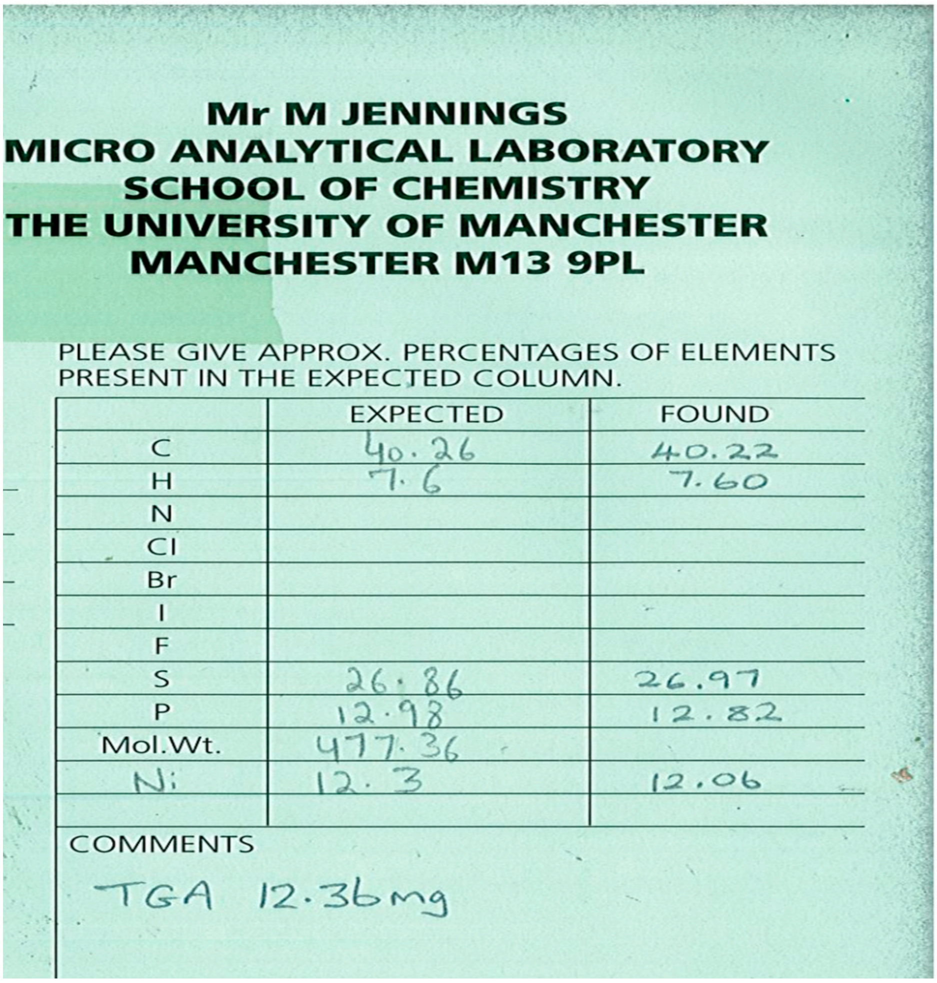

Mr M JENNINGS
MICRO ANALYTICAL LABORATORY
SCHOOL OF CHEMISTRY
THE UNIVERSITY OF MANCHESTER
MANCHESTER M13 9PL

PLEASE GIVE APPROX. PERCENTAGES OF ELEMENTS PRESENT IN THE EXPECTED COLUMN.

	EXPECTED	FOUND
C	40.26	40.22
H	7.6	7.60
N		
Cl		
Br		
I		
F		
S	26.86	26.97
P	12.98	12.82
Mol.Wt.	477.36	
Ni	12.3	12.06

COMMENTS

TGA 12.36mg

Figure S1. Elemental analysis of [Ni(iBu$_2$PS$_2$)$_2$].

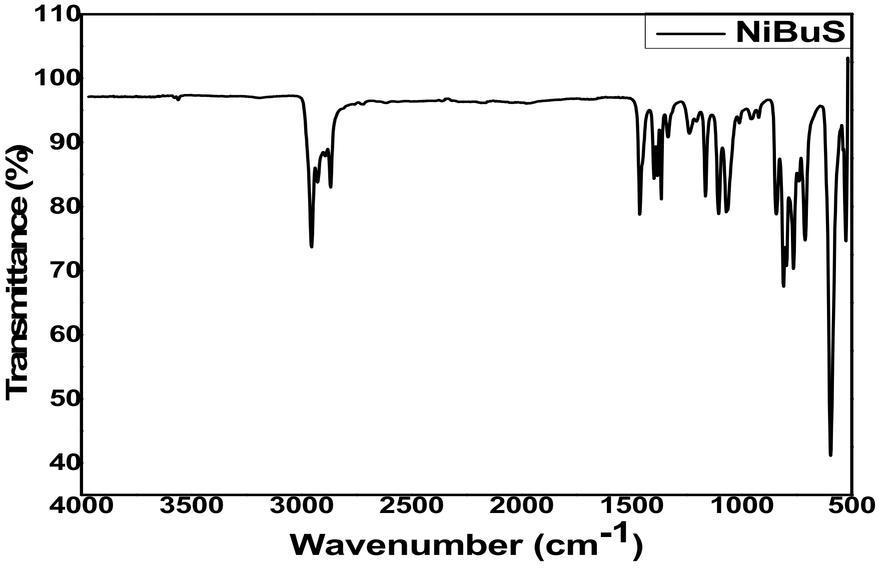

Figure S2. FTIR of $[Ni(iBu_2PS_2)_2]$.

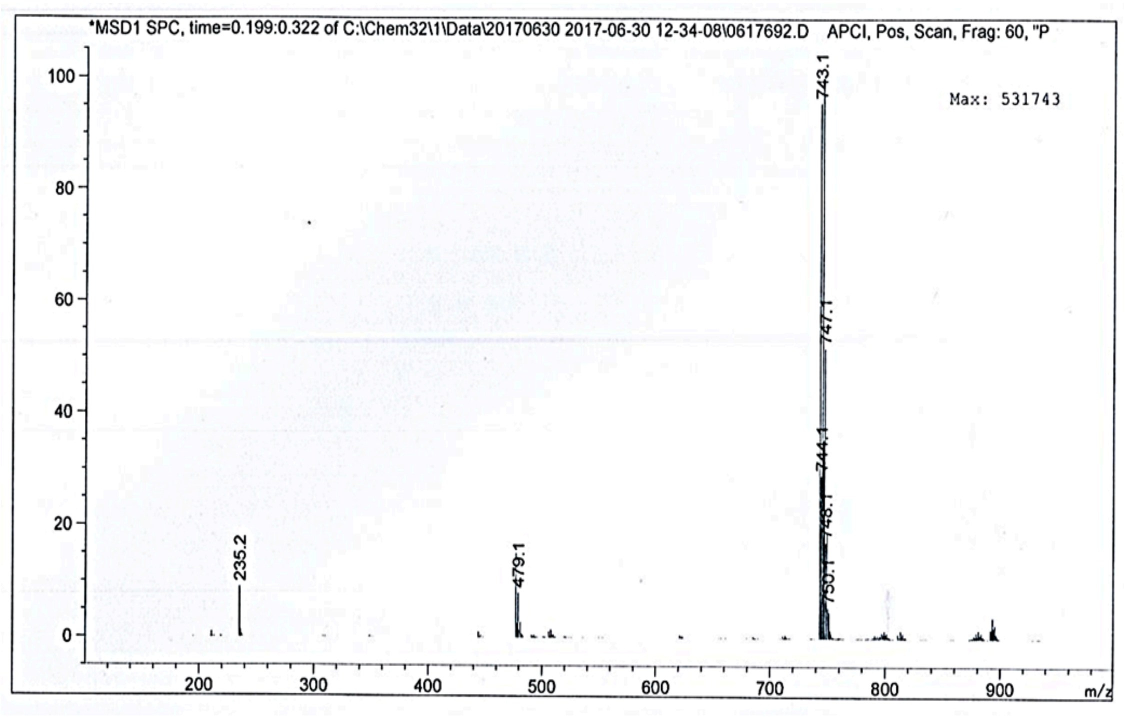

Figure S3. Mass Spectra of $[Ni(iBu_2PS_2)_2]$.

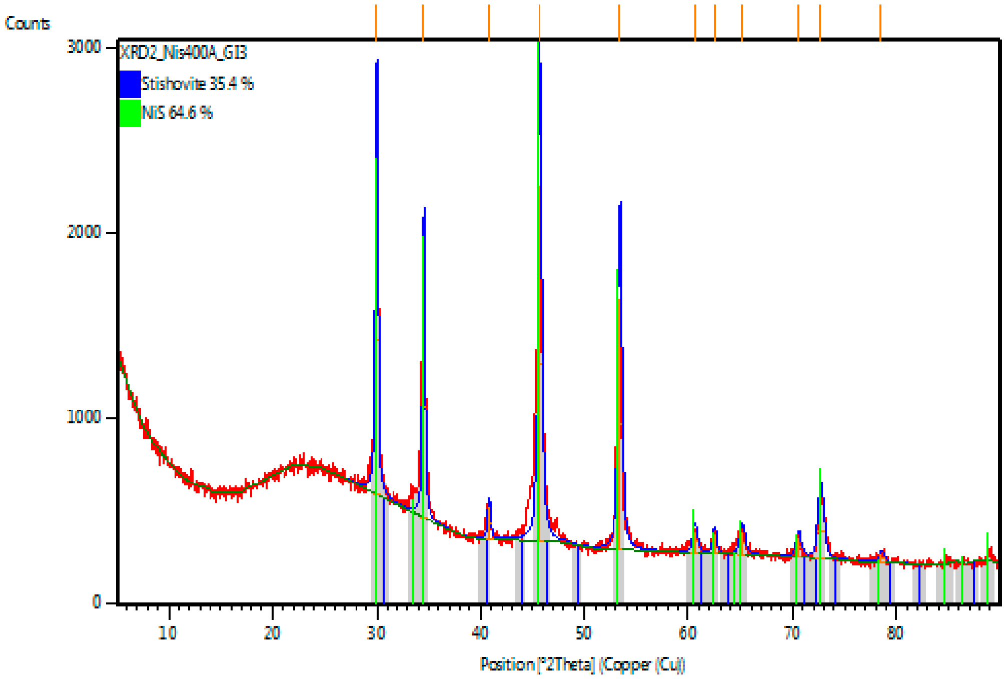

Peak List

Pos.[°2Th.]	Height [cts]	FWHMLeft[°2Th.]	d-spacing [Å]	Rel. Int. [%]	TipWidth	Matched by
29.9883	1657.23	0.2952	2.97981	86.17	0.3542	96-900-9241
34.4906	1206.79	0.2952	2.60044	62.75	0.3542	96-900-9241
40.7825	172.01	0.2952	2.21261	8.94	0.3542	96-900-7153
45.6720	1923.14	0.4428	1.98647	100.00	0.5314	96-900-9241
53.3815	1358.14	0.4428	1.71634	70.62	0.5314	96-900-9241
60.6115	114.10	0.4920	1.52777	5.93	0.5904	96-900-7153;96..
62.4412	105.87	0.3936	1.48734	5.51	0.4723	96-900-9241
65.1305	117.59	0.5904	1.43228	6.11	0.7085	96-900-9241
70.5385	101.38	0.5904	1.33514	5.27	0.7085	96-900-7153;96..
72.6745	302.45	0.5904	1.30108	15.73	0.7085	96-900-7153;96..
78.5452	50.47	0.5904	1.21789	2.62		

0.7085 96-900-7153;96..

Pattern List

Visible	Ref.Code	Score	Compound Name	Displ.[°2Th]	Scale Fac.	Chem. Formula
*	96-900-7153	16	Stishovite	0.000	0.106	Si2.00 O4.00
*	96-900-9241	78	NiS	0.000	1.042	Ni2.00 S2.00

Graphics

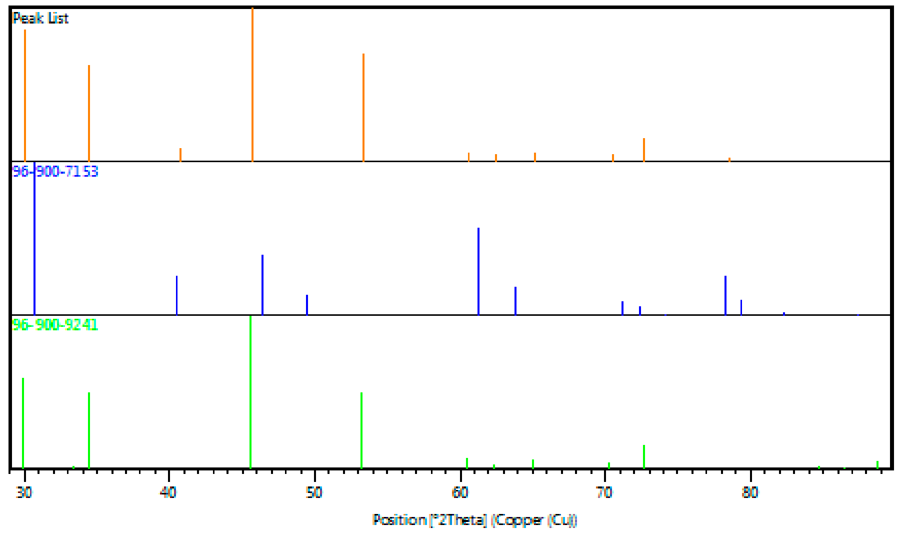

Figure S4. Rietveld Refinement graphics of NiS.400A

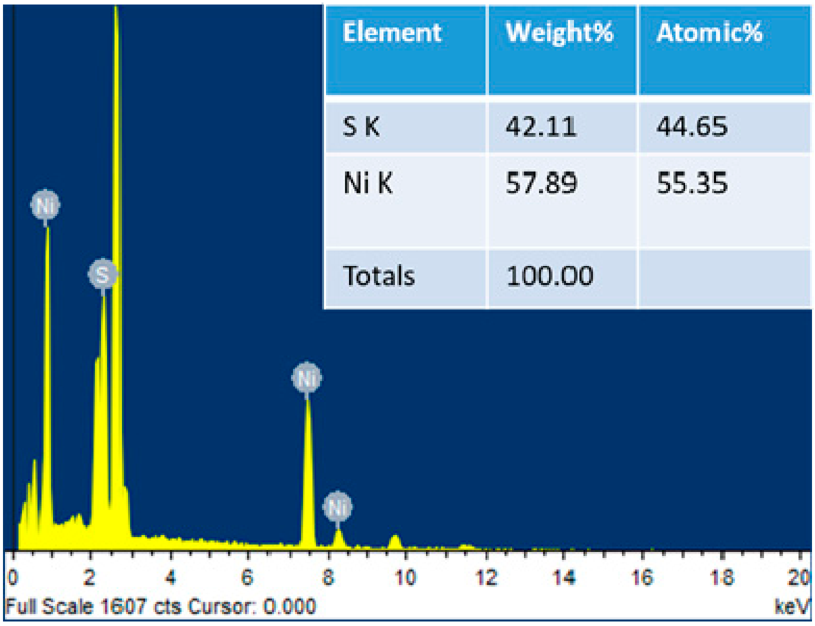

Element	Weight%	Atomic%
S K	42.11	44.65
Ni K	57.89	55.35
Totals	100.00	

Figure S5a. EDX analysis of NiS at 400 °C

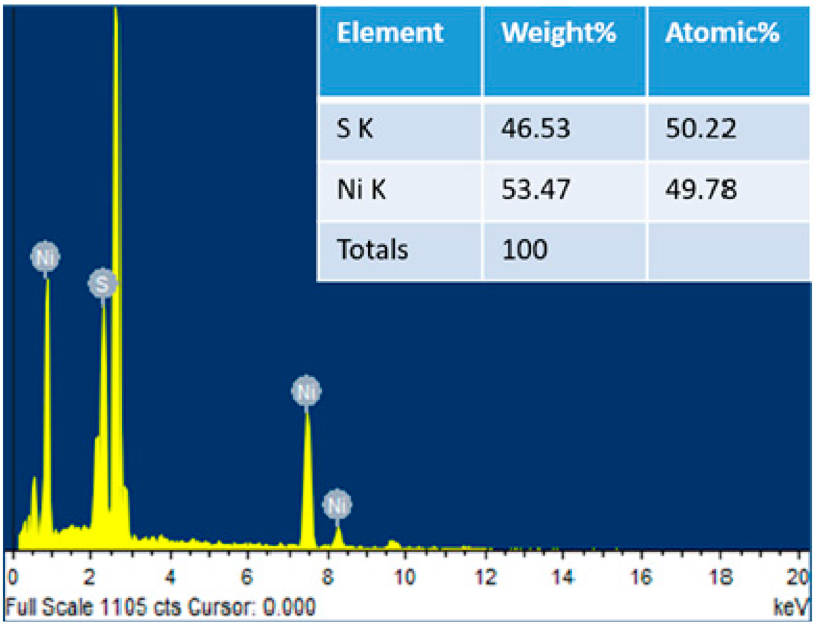

Element	Weight%	Atomic%
S K	46.53	50.22
Ni K	53.47	49.78
Totals	100	

Figure S5b. EDX analysis of NiS at 450 °C
